# The effect of railways on bird diversity in farmland

**DOI:** 10.1007/s11356-019-06245-0

**Published:** 2019-08-27

**Authors:** Joanna Kajzer-Bonk, Piotr Skórka, Maciej Bonk, Magdalena Lenda, Elżbieta Rożej-Pabijan, Marta Wantuch, Dawid Moroń

**Affiliations:** 1 Department of Entomology, Institute of Zoology and Biomedical Research, Faculty of Biology, Jagiellonian University, Gronostajowa 9, 30-387 Kraków, Poland; 2 Institute of Nature Conservation, Polish Academy of Sciences, Mickiewicza 33, 31-120 Kraków, Poland; 3 Australian Research Council Centre of Excellence for Environmental Decisions, School of Biological Sciences, The University of Queensland, QLD, St. Lucia, 4072 Australia; 4 Institute of Biology, Pedagogical University of Cracow, Podchorążych 2, 30-084 Kraków, Poland; 5 Institute of Environmental Sciences, Jagiellonian University, Gronostajowa 7, 30, –387 Kraków, Poland; 6 Institute of Systematics and Evolution of Animals, Polish Academy of Sciences, Sławkowska 17, 31-016 Kraków, Poland

**Keywords:** Agriculture, Biodiversity, Man-made habitat, Linear structure, Railroad, Tracks

## Abstract

With a length exceeding 210,000 km in Europe, railways are common linear features dissecting landscapes. However, the impact of railway networks on biodiversity is equivocal. In this study, we investigated the effect of railway embankments on bird diversity components in an agricultural landscape in southern Poland. Forty transects including 20 along railways and 20 as controls in open fields were established. Birds were counted twice in 2009, and environmental characteristics were estimated for each transect. Ordination techniques and generalized additive models were used to compare species composition, richness, abundance, conservation status, population trends and phylogenetic and functional diversity indices between railway and field transects. Species richness and phylogenetic diversity but not abundance nor functional diversity were higher along railway transects than along field transects. Diversity indices near railways, mostly species richness and phylogenetic diversity, were positively associated with bush cover, wet meadow cover, wetland cover and the slope of the railway but negatively associated with dry meadow cover and field cover. Our study shows that railway embankments may be beneficial for bird diversity but probably do not alter the functional properties of bird communities as much as open fields. Proper management of these linear habitats may increase their value for birds and contribute to long-term bird community persistence.

**Electronic supplementary material:**

The online version of this article (10.1007/s11356-019-06245-0) contains supplementary material, which is available to authorized users.

## Introduction

Habitat destruction, pollution, agriculture intensification and invasions of alien species deeply impact biodiversity and the functioning of ecosystems (McKee et al. 2003; Erwin 2008; Cardinale et al. 2012; Moroń et al. 2012). Many species are able to adapt to human-modified environments, but species unable to respond favourably to these environmental changes become extinct, or their populations diminish (Erwin 2008; Miraldo et al. 2016). With declining species richness and abundance, species-specific characteristics as well as all communities may be disturbed. Thus, the loss of species diversity is associated with alterations in phylogenetic and functional diversity (Pan et al. 2016).

In landscapes dominated by human activity, linear structures are among the key factors affecting animal and plant population functioning (Forman et al. 2003; Benítez-López et al. 2010). A linear structure is any elongated landscape feature (a verge, embankment, hedge, tree and/or bush row, ditch) which is usually situated along a transportation line (a road or railway track), differs from adjacent habitat and diversifies the landscape. The role of linear structures is ambiguous, with prevailing reports of their negative impact on biodiversity (Borda-de-Água et al. 2017; Barrientos et al. 2019), and there is a substantial disproportion in the number of studies concerning effects of roads rather than railways (Popp and Boyle 2017). The presence of transportation lines in a landscape seems to be an obvious barrier for low-mobility organisms leading to the fragmentation of populations (Andrews 1990; Fahrig 2003), emergence of edge effects (Forman et al. 2003) and decline in genetic variation (Balkenhol and Waits 2009; Holderegger and Di Giulio 2010). Several animal species avoid crossing transportation lines (Ries and Debinski 2001; Skórka et al. 2013a) because dispersal and movement pose increased mortality risk (Nowicki et al. 2014; Skórka et al. 2013b). Road and railway mortality affect both good dispersers such as birds and mammals (Benítez-López et al. 2010; Karlsson et al. 2007; Silva et al. 2012) and poor dispersers such as small-bodied herpetofauna (Fahrig and Rytwinski 2009; Santos et al. 2017) and insects (Askling and Bergman 2003; Tamayo et al. 2015; Skórka et al. 2013b). Moreover, transportation lines may work as ecological traps by attracting organisms preferring bare, warm surfaces (Stevens et al. 2006) or by trapping organisms between rails without access to water and food (Budzik and Budzik 2014, but see Kaczmarski and Kaczmarek 2016).

However, linear structures do not only affect population functioning and biodiversity negatively. An example of a positive effect is wheel ruts serving as important habitats for some amphibians (Babik and Rafiński 2001; Cayuela et al. 2015). Birds may use linear structures as breeding habitat due to their lower predatory pressure, higher temperature and larger number of food items, including road-killed ones (Mumme et al. 2000; Morelli 2013; Morelli et al. 2014; Heske 2015; but see Vierling (2000) which revealed sink effect of roadside ditches). Despite a lot of casualties (Carvalho et al. 2017; Godinho et al. 2017a; Lucas et al. 2017; Murias et al. 2017; Santos et al. 2017), some animal groups such as raptors and scavengers may be highly dependent on road habitats as sources of food (Benítez-López et al. 2010; Coleman and Fraser 1989). Linear structures change local abiotic conditions, leading to the emergence of strong environmental gradients that may increase the availability of niches and thus increase species diversity (Amarasekare 2003; Nord and Forslund 2015). Previous studies revealed that a landscape mosaic ensures persistence and higher abundances of rare and endangered species (Atauri and de Lucio 2001; Pöyry et al. 2005; Kajzer-Bonk et al. 2016). In homogenous agricultural landscapes, linear structures may contribute to landscape complexity (Morelli et al. 2015; Villemey et al. 2018). The presence of verges, embankments or ditches alongside roads and railways provides a diversity of trees, bushes and herbaceous plants, which may include a significantly higher number of native plants compared to surrounding areas (Forman et al. 2003; Deckers et al. 2005) and provide habitat for many pollinating insects (Skórka et al. 2013b; Moroń et al. 2014). In homogenous intensive agricultural landscapes, such features may be the only locations where species survive (Wynhoff et al. 2011). Van Geert et al. (2010) and Moroń et al. (2017) revealed that such linear habitats may function as biological corridors by facilitating the dispersal of insects and insect-pollinated plants. Railway embankments may increase the alpha diversity and community turnover of invertebrates and plant taxa (Moroń et al. 2014, 2017; Vandevelde and Penone 2017). From four studied linear structure types (roads, railways, transportation bridges and rivers) railways were revealed as the most permeable for songbirds movements (Tremblay and Clair 2019).

Despite an increasing number of findings concerning railway ecology, it is relatively novel discipline with overwhelming number of research reporting negative effects on biodiversity (Barrientos and Borda-de-Água 2017; Godinho et al. 2017a; Malo et al. 2017; Murias et al. 2017; Santos et al. 2017; Barrientos et al. 2019) and effects of railways on bird biology and bird communities are rarely studied. For example, Popp and Boyle (2017) found altogether 3 vs. 62 papers concerning railway and road effects on birds, respectively. The high diversity of plants and invertebrates on railway embankments (Moroń et al. 2014) suggests that such locations have a rich food base attractive for birds. Railway proximity decreases vigilant behaviour of snowfinches (Ge et al. 2011) and increases the abundance of seven ground-dwelling bird species in Tibet (Li et al. 2010). Electrical lines and pylons associated with railways may act as substitutes for some natural elements (e.g. old trees) that may be perching, singing and resting sites but are currently frequently removed from agricultural landscapes (Morelli et al. 2014; Tryjanowski et al. 2014; but see Carvalho et al. 2017). Moreover, train traffic is usually much lower than road traffic, suggesting low vehicle-related mortality (Morelli et al. 2014). Thus, railway embankments may be suitable habitats for birds, especially in altered, homogenous agricultural landscapes.

The aim of this study was to compare bird diversity and community composition along railway line transects and control transects located in agricultural landscapes. We hypothesized that linear structures along railways may reinforce species diversity in the landscapes. Specifically, we predicted that an agricultural landscape with a railway would have a more diverse bird community in terms of (1) taxonomy, (2) phylogeny, (3) functionality and 4) conservation status than would a landscape without railways.

## Materials and methods

### Study area

**Fig. 1:**
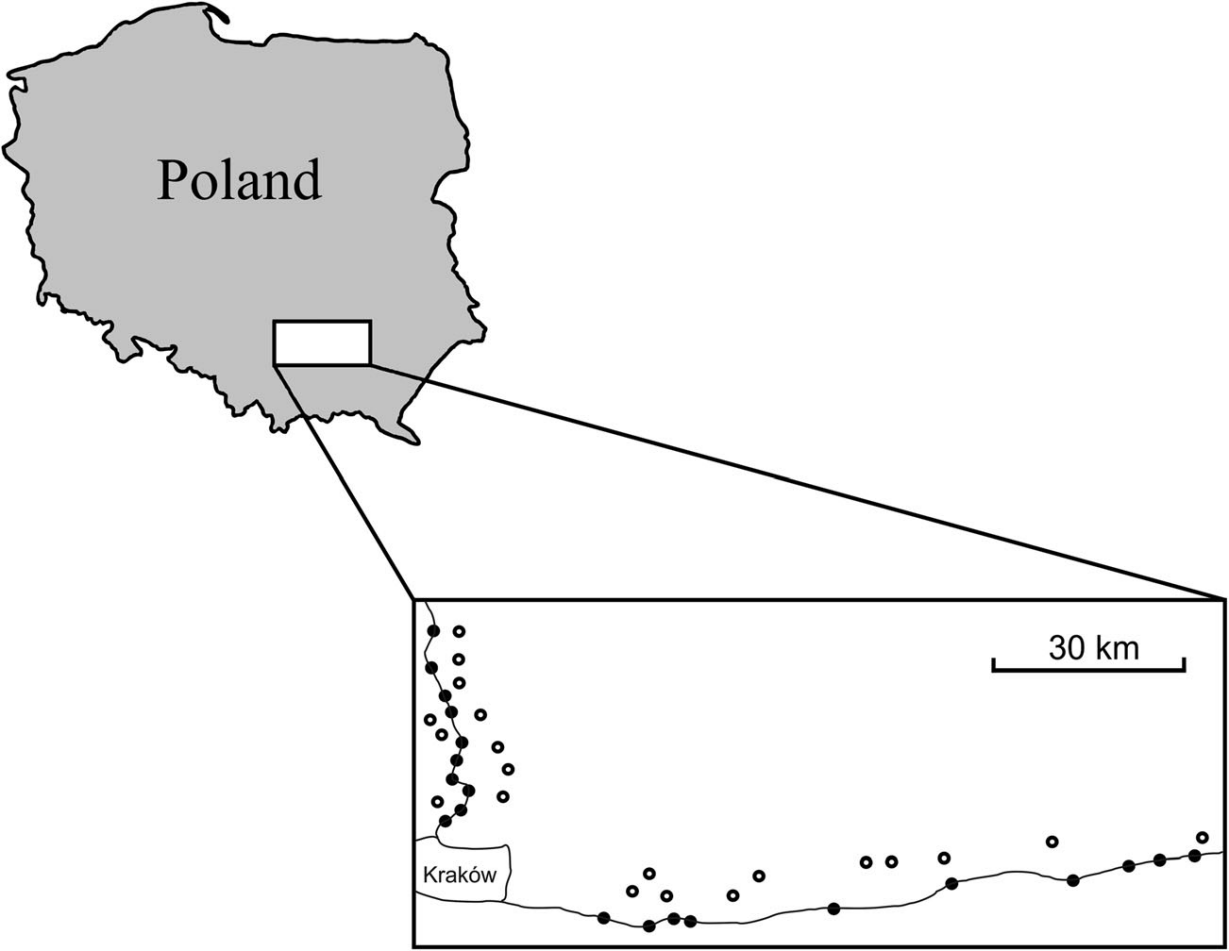
Map of the study area. Locations of railway and control transects (black and empty dots, respectively), and railways (solid lines) are shown

The study was conducted in 2009 in the Małopolska region in southern Poland. Data were collected in an agricultural landscape along 40 randomly selected transects 1 km long and 50 m wide located at least 2 km away from each other (Fig. 1). Twenty of the transects were situated along the railway, and the remaining 20, in landscapes similar in terms of use but without railways. Two surveys during the breeding season (first in May and following in June) in early morning and in fine weather conditions (no strong wind or rain) were performed to assess bird occurrence and abundance. Survey was conducted by one observer walking along a transect with a velocity of 2 km/h and counting visible and/or singing birds in a 50-m buffer, including specimens flying over at low altitude (below 50 m), e.g. foraging swallows and gulls and excluding birds flying high above transects (> 50 m) in a directional way (vagrants, migrants). There was a balance in terms of survey order: transects were equally sampled during different times of the morning. It took three days to complete one survey. Bird counts within a survey were done during consecutive days. Surveys usually started an hour since sunrise and ended at 11 a.m. Along each transect, the cover of land types (meadows, arable fields, buildings, wetland, fallow land, and trees/bushes) in a 50-m buffer, slope, transect cardinal direction and “food availability index” expressed as the number of butterflies which is potentially a good predictor of bird richness and abundance (Skórka et al. 2010) were assessed. Both adult butterflies and their larvae are important food for many bird species (e.g. Marciniak et al. 2007). Even passerine birds from true finch (Fringillidae) and bunting (Emberizidae) families, which are adapted to eating seeds, feed their offspring with high-protein food composed mainly of insects, including caterpillars and imagoes of butterflies (Holland et al. 2006). Abovementioned variables were estimated using two 25-m-width belts on both sides of the transect and the percent cover of each predictor was quantified every 200 m. The crops were low (up to a 1-m height) and there dominated cereals (wheat, rye, oat), root crops (potatoes), oil crops (rapeseed) and vegetables (cabbage, parsley, tomatoes) and did not preclude visibility in both in railway and control field transects. There was no cornfield in the area during our study. Railway transects ran exactly on track and buffer describing landscape type excluded area of railway track. There were no additional linear structures in control transects (i.e. roads with associated windbreaks). This procedure ensured focusing on railway effect only. Additionally, forest cover in a 500-m radius of each transect as only non-open habitat diversifying agricultural landscape was measured using aerial photographs acquired from Google Earth program and ImageJ software (Abramoff et al. 2004).

### Data handling

Nine measures of bird diversity were calculated separately for each transect: three related to taxonomic diversity and distribution, two related to phylogenetic diversity and four related to functional diversity. Additionally, we calculated two indices of conservation status.

Species richness, total abundance and a commonness index were taxonomic diversity indices. The maximal number of recorded bird species and individuals over two surveys along each transect were proxies of species richness and abundance . The commonness index was the average total population size in Poland estimated for each species and averaged over the species recorded along a given transect. Population estimates for the years 2008–2012 were used (Chodkiewicz et al. 2015).

Two indices of phylogenetic diversity were used: Faith’s standardized phylogenetic distance between taxa and their evolutionary distinctiveness (Frishkoff et al. 2014; Isaac et al. 2007). These indices were independent of the number of species. The bird phylogenetic tree (Jetz et al. 2012) was built online (http://birdtree.org; Fig. S1 in Supplementary material 1) and it was used to calculate the phylogenetic diversity indices via the package “picante” (Kembel et al. 2010) in R (R Core Team 2017).

Four indices of functional diversity were used: functional richness, functional evenness, functional divergence and functional dispersion. These metrics are based on species traits and describe the functional dimension of biodiversity (de Bello et al. 2010). We used avian traits linked with life histories, breeding, foraging and dispersal biology (Huang et al. 2015; Morelli et al. 2017; Storchová and Hořák 2018, Table S1). Functional diversity is related to taxonomic diversity and often predicts community assembly rules (e.g. productivity and resistance to disturbance) better than does species richness (Mouchet et al. 2010). Traits used in the calculations were coded as 39 variables (Table S1 contains all variables and the levels considered for each): body mass, brain mass, sexual dimorphism, lifespan, clutch size, age at first reproduction, incubation time, number of broods per year, mean egg mass, length of incubation period (days), length of fledging period (days), life span, migration mode (long-distance migrant, short-distance migrant, facultative migrant, sedentary), food categories (frugivore, folivore, granivore, invertebrates, fish, omnivore or carrion), mode of development (precocial, semi-precocial, semi-altricial, or altricial) and sociality during the breeding season (solitary, semi-colonial, or colonial). Sexual dimorphism, development and migration modes, food categories and sociality were coded as categorical binary variables (e.g. whether a species was colonial was coded as either 0 or 1). This approach allowed us to include plasticity in species traits (e.g. the rook, *Corvus frugilegus*, forages on both arthropods and seeds of various plants, (Czarnecka and Kitowski 2010)) in the analyses. Functional richness was calculated as the volume of a multidimensional space with traits of species in their assemblage (Villéger et al. 2008). Functional evenness represented the uniformity of the species abundance distribution across the volume of characteristics. Functional divergence expressed the extent to which species abundances were on the limits of the functional space after accounting for the functional richness (Villéger et al. 2008; Mouchet et al. 2010). If the most abundant species have dissimilar traits then they weakly compete and thus functional divergence has high values. Functional dispersion shows the spread or variability in the presence of species and is less sensitive to outliers and independent of species richness (Laliberté and Legendre 2010). The functional diversity indices were weighted by species abundance. We calculated these indices in the “FD” package of R (Laliberté et al. 2015).

Two indices of conservation status were used: a modified category of IUCN conservation status (IUCN 2018) and the proportion of declining species. To calculate the mean IUCN category, we used the following scale: 1—species of least concern with an increasing population size, 2—species of least concern with a stable population size, 3—species of least concern with a decreasing population size, 4—species of least concern with an unknown population trend, 5—near-threatened species (with a decreasing population size), and 6—vulnerable species (with a decreasing population size). There were no species considered endangered or critically endangered. Each species in a community was scored, and the mean value for each habitat was calculated.

To calculate the proportion of declining species, we used IUCN data (IUCN 2018). Each species was scored based on whether its population size is decreasing or increasing/stable (scored 1 and 0, respectively; Table S2 in Supplementary material 1). The number of declining species divided by the total number of species recorded along a given transect was used in the analyses.

### Statistical analysis

Bird species composition and abundance between railway line and field control transects were compared by using non-metric multidimensional scaling (NMDS) implemented in the “vegan” package (Oksanen et al. 2013) in R. We compared the distribution of loadings of abundance counts along the first three NMDS axes and the statistical significance with the permutation test (999 permutations). Permutational multivariate analysis of variance (PERMANOVA) was used to find differences in centroids and dispersion of the groups representing two habitats. Moreover, we identified species that were characteristic of railway line and control transects in farmland by using Indicator Species Analysis in the “indicspecies” package (de Caceres and Legendre 2009) in R. The strength of association between species and habitat type was checked by the permutation test (999 permutations).

We compared the nine measures of bird diversity and two metrics of conservation status between railway and farmland transects. We used generalized additive models (GAMs) with Poisson (species richness), negative binomial (bird abundance) and Gaussian (the remaining indices) error distributions implemented in “mgcv” package (Wood 2006) in R (R Core Team 2017). In the GAMs, geographical latitude and longitude were fitted as the interaction of regression splines to control for the spatial autocorrelation of the dependent variables (Wood 2006). We used GAMs to test for effects of the environmental variables on biodiversity indices and bird conservation status near railway lines. The ten studied environmental variables were the cover (measured in a 50-m buffer) of arable fields, dry meadows, wet meadows, fallow land, wetland, buildings and bushes; forest cover within 500 m; slope of the embankments; “food availability index” and cardinal direction of the line. There were significant correlations between the continuous explanatory variables (Fig. S1 in Supplementary material 1). Thus, we used a principal component analysis (PCA) with varimax rotation to transform the 10 continuous variables into four orthogonal components (Fig. S1 in Supplementary material 1). Associations between the four extracted components and original explanatory continuous variables are given in Table 1. The four extracted components and cardinal direction of the railway transect (E, N, NE and NW) were thereafter used in the additive models.

**Table 1.** Principal component analysis (PCA) based on the 10 continuous explanatory variables. Loadings higher than 0.4 are in italic

Variable	PCA1	PCA2	PCA3	PCA4
Bush cover	*− 0.576*	*− 0.627*	*−* 0.009	0.248
Dry meadow cover	*0.656*	0.005	*− 0.629*	0.287
Wet meadow cover	*− 0.764*	0.067	*−* 0.162	*− 0.433*
Field cover	*0.626*	0.350	*0.631*	*−* 0.009
Wetland cover	*− 0.808*	0.108	*−* 0.049	*−* 0.254
Building cover	0.308	*− 0.566*	*−* 0.127	*− 0.664*
Fallow land cover	0.385	*−* 0.019	*0.472*	*− 0.441*
Slope	*− 0.744*	*0.507*	0.251	0.170
Food availability index	0.029	*0.696*	*− 0.434*	*−* 0.274
Forest cover in a 500 m radius	*−* 0.306	*−* 0.303	0.310	0.221
Variance explained (%)	33	17	14	12

## Results

**Fig. 2:**
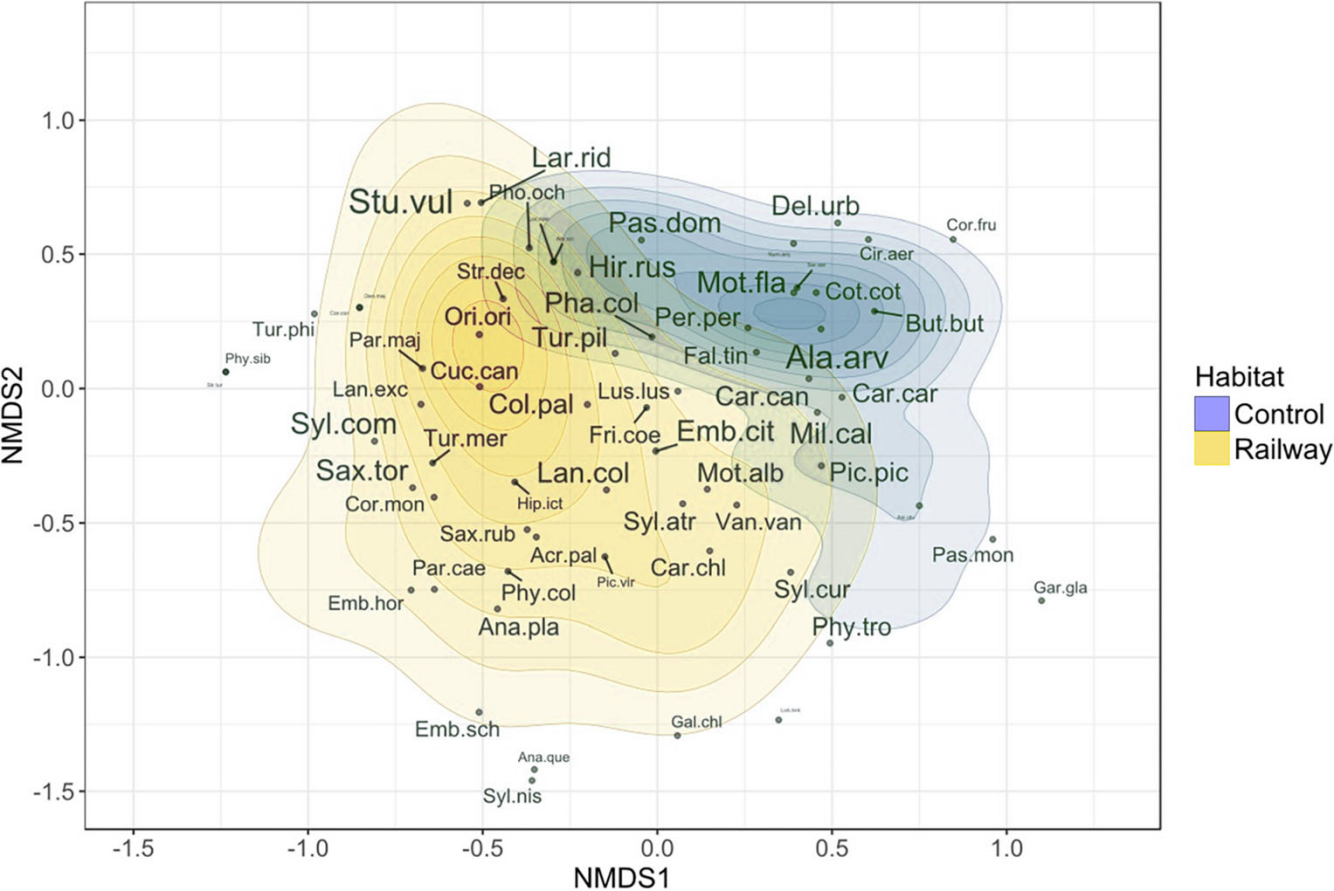
Dissimilarities between bird communities along railway line (yellow) and farmland control (blue) transects depicted via kernel density estimates of site-specific scores of species along the two first axes from the non-metric multidimensional scaling (NMDS) analysis. Sizes of species labels are proportionally scaled to the total abundance of the species and are explained in Table S1

In total, we recorded 1 644 individuals of 67 bird species along the railway and farmland control transects (Table S2). Altogether, 18, 17, 27, 2, 2 and 1 species were found in each category of IUCN status, ordered with increasing vulnerability (see methods for more details and Table S2 in Supplementary material 1). Along the railway transects, 58 species (923 individuals) were recorded, and 50 species (721 individuals) were recorded along control transects (Table S2). The most common species was the starling *Sturnus vulgaris*, with 290 individuals, followed by the skylark *Alauda arvensis*, with 193 individuals, and the common whitethroat, *Sylvia communis*, with 72 individuals. The NMDS analysis (non-metric fit *R*^2^ = 0.954, stress = 0.190) showed that the bird community near railway lines was significantly different (PERMANOVA *F* = 5.863; df = 1, 39; *P* = 0.005, *R*^2^ = 0.15) from that recorded along control field transects: the two point clouds representing the two transect types showed little overlap (Fig. 2). In total, 17 species were present only along railway transects, while nine species were present only along field control transects (Table S2).

Seven species were characteristic of railways as it was revealed by the Indicator Species Analysis. These species were: the common stonechat, *Saxicola torquatus* (*rubicola*) (estimate = 0.879, *P* < 0.001); the common whitethroat, *Sylvia communis* (estimate = 0.797, P = 0.001); the common chaffinch, *Fringilla coelebs* (estimate = 0.617, *P* = 0.021); the golden oriole *Oriolus oriolus* (estimate = 0.609, *P* = 0.029); the common chiffchaff, *Phylloscopus collybita* (estimate = 0.548, *P* = 0.022); the mallard, *Anas platyrhynchos* (estimate = 0.500, *P* = 0.048); and the common reed bunting, *Emberiza schoeniclus* (estimate = 0.500, *P* = 0.048). Only one species, the common quail, *Coturnix coturnix* (estimate = 0.586, *P* = 0.021), was selected as an indicator species for the field control transects.

**Fig. 3:**
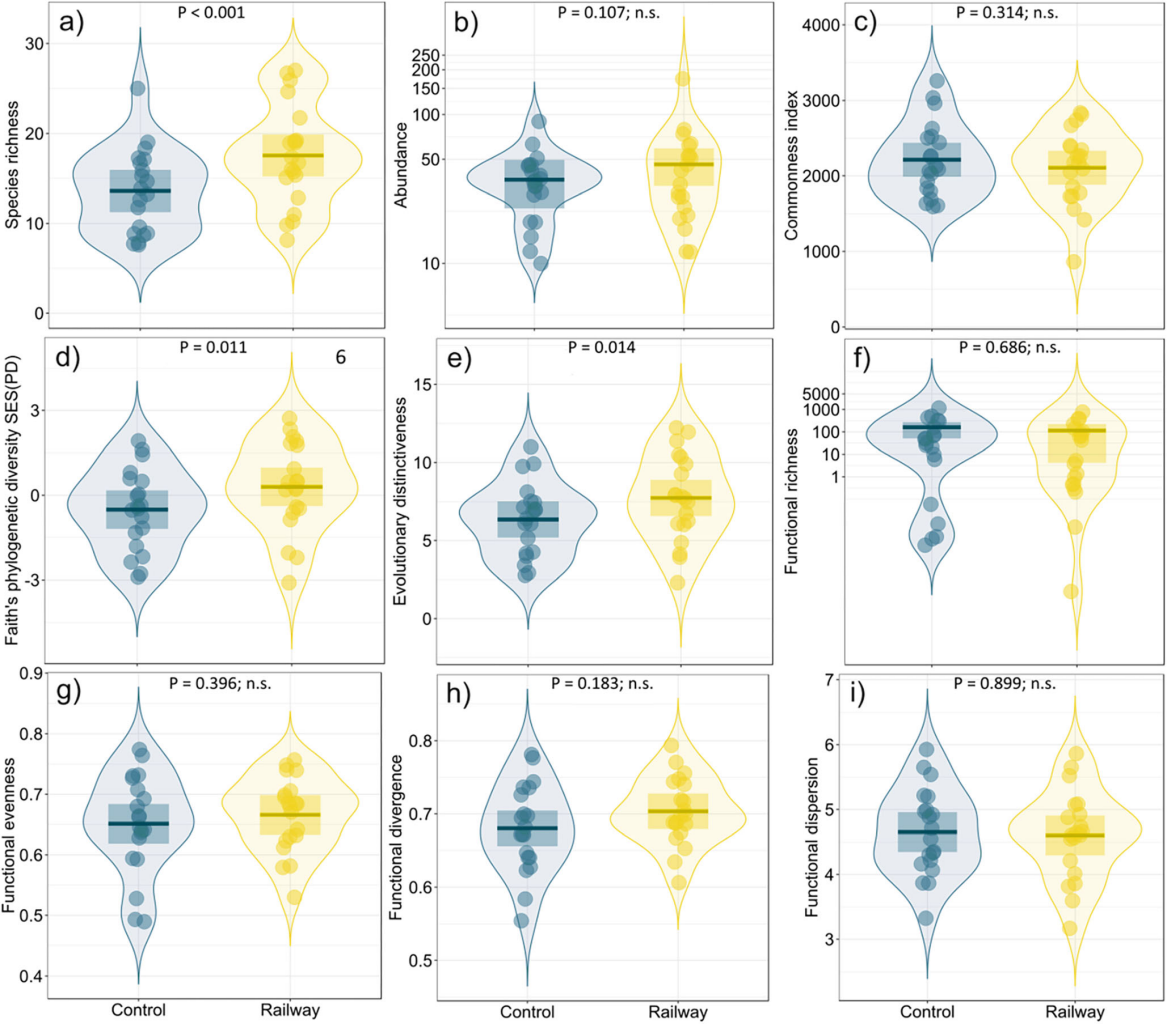
The comparison of nine bird diversity indices between railway (yellow) and farmland (blue) transects. Boxplots show means (horizontal lines) and 95% confidence intervals (rectangles). The density of points (violins) is also shown. Graphs for abundance and functional richness have a logarithmic *y*-axis. Explanations: *n.s.* statistically non-significant difference

**Fig. 4:**
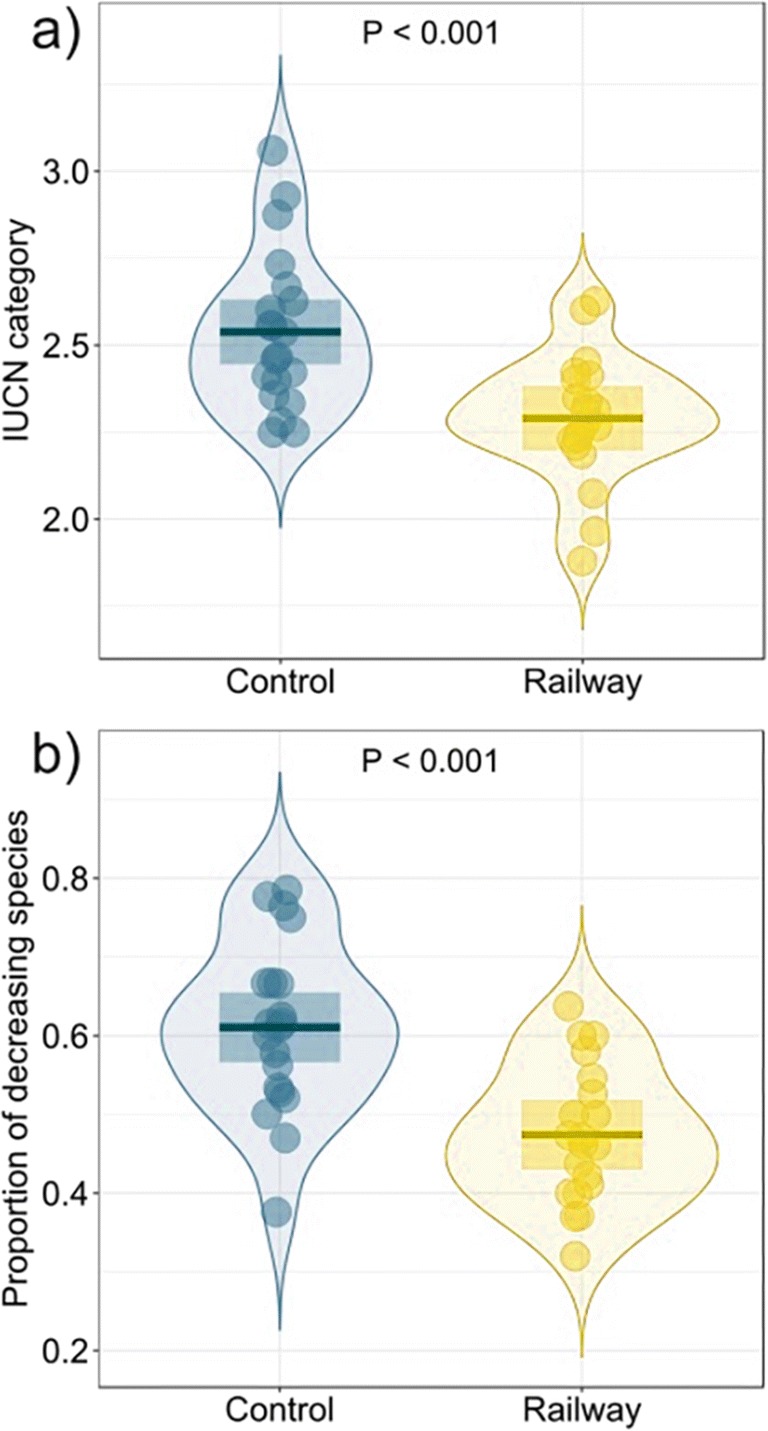
The comparison of conservation indices between railway (yellow) and farmland (blue) transects. Explanations: see Fig. 3

Compared to the control transects, the railway transects hosted a higher number of species but a similar abundance of birds (Fig. 3, Table 2). Species occurring along both transect types were similarly common in Poland (Fig. 3, Table 2). Faith’s standardized phylogenetic diversity and evolutionary distinctiveness were higher along railway transects than along farmland transects (Fig. 3, Table 2). All examined functional diversity indices had similar values in railway and field transects (Fig. 3, Table 2). However, the conservation status measured as mean IUCN category and the proportion of declining species were lower along railway line transects than along control field transects (Fig. 4, Table 2).

**Table 2:** The formal tests (generalized additive models) comparing bird diversity components and bird conservation status between railway and farmland transects. Estimates with standard errors (in brackets), test statistic (chi-square for species richness and abundance, *F* otherwise), variance explained (*R*^2^_adj_), and *P* values are given. Statistically significant differences have italic *P* values

Explanatory variables	Intercept	Transect type: railway*	Statistic	*R* ^2^_adj_	*P*
Species richness	2.578 (0.061)	0.282 (0.081)	12.11	0.48	*< 0.001*
Abundance	3.509 (0.095)	0.214 (0.133)	2.591	0.37	0.107
Commonness index	2242.1 (111.0)	*−* 163.8 (160.3)	1.045	0.03	0.314
Faith’s phylogenetic diversity	*−* 0.606 (0.257)	0.983 (0.364)	7.265	0.44	*0.011*
Evolutionary distinctiveness	6.232 (0.441)	1.616 (0.626)	6.664	0.43	*0.014*
Functional richness	151.630 (40.090)	*−* 28.350 (69.630)	0.166	0.17	0.686
Functional evenness	0.648 (0.016)	0.020 (0.023)	0.738	0.04	0.396
Functional divergence	0.680 (0.012)	0.024 (0.017)	1.847	0.02	0.183
Functional dispersion	4.615 (0.128)	*−* 0.023 (0.184)	0.016	0.28	0.899
IUCN conservation category	2.541 (0.046)	*−* 0.254 (0.066)	14.910	0.25	*< 0.001*
Proportion of declining species	0.611 (0.022)	*−* 0.139 (0.032)	19.300	0.30	*< 0.001*

*Control transects in farmland were the reference category, i.e. control = 0

Additive models showed that species richness was negatively correlated with PCA1 (and thus positively correlated with bush cover, wet meadow cover, wetland cover and the slope of the railway but negatively correlated with dry meadow cover and field cover, Table 3). The abundance of birds was negatively correlated with PCA2 (and thus positively correlated with bush cover and building cover but negatively correlated with slope and the food availability index), PCA3 (and thus positively correlated with dry meadow cover and food resources but negatively correlated with field cover and fallow land cover) and PCA4 (and thus positively correlated with wet meadow cover, fallow land cover and building cover, Table 3). The effects of environmental variables on Faith’s standardized phylogenetic diversity and evolutionary evenness were similar to that on species richness (Table 3). Not one environmental variable was associated with the commonness index nor with the functional diversity indices (Table 3). Mean IUCN category was negatively associated with PCA4 (and thus positively associated with wet meadow cover, fallow land cover and building cover (Table 3). The proportion of declining species was positively correlated with PCA1 (and thus negatively correlated with bush cover, wet meadow cover, wetland cover and the slope of the railway embankment but positively correlated with dry meadow cover and field cover, Table 3). The proportion was also lower along railway lines directed N-S than along those oriented E–W (Table 3).

**Table 3:** The effect of environmental variables on bird diversity and conservation indices near railway lines. Generalized additive model estimates of slopes of functions and their standard errors (in brackets) are presented. Statistically significant effects are italic: ***P < 0.001, **P < 0.01, and *P < 0.05. See also Table 1 for an explanation of the principal components (PCA1–PCA4) used in these analyses

Models for:	Explanatory variables						
	Intercept	PCA1	PCA2	PCA3	PCA4	Cardinal direction	*R* ^2^_adj_
Species richness	2.38 (0.26)***	− 0.16 (0.07)*	*−* 0.02 (0.08)	0.02(0.05)	0.01 (0.05)	N **=** 1.01 (0.54) NE **=** 0.84 (0.46) NW **=** 0.82 (0.50)	0.82
Abundance	2.03 (0.47)***	*−* 0.01 (0.07)	− 0.23 (0.08)**	− 0.14 (0.04)***	− 0.11 (0.05)*	N **=**3.17 (0.93)*** NE **=**3.40 (0.92)*** NW **=**3.01 (0.93)**	0.90
Commonness	2603.81 (579.39)**	228.08 (125.29)	*−* 156.07 (163.4)	81.61 (96.90)	30.31 (107.50)	N **=***−* 1007.5 (1214.6) NE **=***−* 942.9 (1103.4) NW **=***−* 1021 (1182.9)	0.17
Phylogenetic diversity	− 2.16 (0.72)*	− 0.73 (0.19)**	*−* 0.02 (0.25)	0.14 (0.15)	0.17 (0.17)	N **=**5.28 (1.57)** NE **=**4.66 (1.30)** NW **=**4.83 (1.48)**	0.79
Evolutionary distinctiveness	4.45 (1.13)**	− 1.13 (0.29)**	*−* 0.04 (0.39)	0.24 (0.23)	0.29 (0.26)	N **=**6.87 (2.46)* NE **=**6.35 (2.03)* NW **=**6.49 (2.32)*	0.84
Functional richness	101.65 (200.32)	*−* 23.84 (52.03)	*−* 19.05 (41.81)	*−* 9.94 (46.11)	*−* 9.94 (46.11)	N **=***−* 25.09 (438.2) NE **=** 124.41 (362.16) NW **=***−* 12.21 (412.23)	0.00
Functional evenness	0.65 (0.07)***	*−* 0.01 (0.02)	0.01 (0.02)	0.02 (0.1)	*−* 0.01 (0.01)	N **=** 0.08 (0.15) NE **=***−* 0.02 (0.12) NW **=** 0.05 (0.14)	0.00
Functional divergence	0.81 (0.07)***	*−* 0.00 (0.01)	0.01 (0.01)	0.02 (0.01)	*−* 0.01 (0.01)	N **=***−* 0.20 (0.14) NE **=***−* 0.24 (0.14) NW **=***−* 0.22 (0.15)	0.19
Functional dispersion	3.98 (0.59)***	*−* 0.05 (0.15)	*−* 0.19 (0.21)	0.03 (0.12)	*−* 0.20 (0.14)	N **=** 1.06 (1.29) NE **=** 1.45 (1.07) NW **=** 1.21 (1.22)	0.27
IUCN conservation category	2.41 (0.12)***	0.02 (0.03)	0.05 (0.04)	0.03 (0.03)	− 0.10 (0.03)**	N **=***−* 0.39 (0.26) NE **=** 0.06 (0.22) NW **=***−* 0.24 (0.25)	0.58
Proportion of declining species	0.61 (0.06)***	0.04 (0.02)*	0.02 (0.02)	0.03 (0.01)	*−* 0.01 (0.01)	**N =** − 0.35 (0.13)* NE **=***−* 0.18 (0.11) **NW =** − 0.29 (0.13)*	0.51

## Discussion

Our study revealed that species richness was higher among railway embankments than along control transects. Consistently, phylogenetic diversity also was higher near railways than in open fields. These results suggest that railways increase taxonomic and phylogenetic richness in agricultural landscapes. This result may be due to the difference in habitat composition, where railway embankments—as important remnants of diverse vegetation—make landscapes locally more of a mosaic and increase the number of available niches in predominantly agricultural landscapes (Coffin 2007; Heikkinen et al. 2004). The ecological role of railways is strongly understudied (Popp and Boyle 2017) despite the fact that the global network of railways is over 1 billion km long (18 429 km long in Poland; International Union of Railways 2015). To date, several studies investigated the effect of railways on bird abundance and richness. There were no differences in wetland bird richness and abundances between study plots adjacent to and far from railways (Godinho et al. 2017b). Similar to our findings, Li et al. (2010) and Wiącek et al. (2015) revealed a higher number of birds in railway proximity compared with control points located far from railways. Some birds habituate and ignore railways probably because railway verges are attractive, increase heterogeneity in homogenous landscape and noise is discontinuous compared with roads (Lucas et al. 2017). As far as other taxa are concerned, several studies have shown (1) a negative effect of railroad due to habitat fragmentation and mortality of large mammals (Ito et al. 2005; Waller and Servheen 2005; Santos et al. 2017), limitation of gene flow in amphibians (Bartoszek and Greenwald 2009) and disturbance (Barrientos et al. 2019); (2) positive effect of railways on pollinator diversity (Moroń et al. 2014) and dispersal (Moroń et al. 2017) as well as (3) a neutral effect on dispersing amphibians (Kaczmarski and Kaczmarek 2016). These results confirm that such human-constructed environments, at least in some cases, may not be harmful or may even be beneficial for biodiversity and should not be neglected in modern nature conservation (Martínez-Abraín and Jiménez 2016; Maclagan et al. 2018).

As our studied transects were located in agricultural landscapes, the potential moderation of functional diversity by railways would be more interesting than that of simple taxonomic diversity. Functional diversity is broadly assumed to be a better predictor of ecosystem productivity and vulnerability than is species diversity (Schleuter et al. 2010). However, there were statistically non-significant differences in all indices of functional diversity between railway transects and open fields, meaning that niches in both types of studied habitats were filled by species with similar features. Also, the abundance of species was similarly distributed in the volume of traits, as indicated by functional divergence and evenness. The lack of differences in functional diversity may stem from proximity and location of control and railway transects in the same agricultural landscape with similar environmental properties. We may not also exclude the possibility that species potential overlapping due to bird fly-overs; however, this effect was probably negligible because only few species such as swallows and gulls exhibited such mobile behaviour. It seems that railways do not introduce new functional properties into agricultural landscapes, but they also do not diminish existing properties, which is also worth noting in light of studies demonstrating the opposite effects in other linear man-made habitats (Fahrig and Rytwinski 2009; Morelli et al. 2015). The effect may also depend on the landscape context. It is possible that in areas with more intense agriculture, the differences in functional diversity would be higher.

Our study indicates that there are some potential ways to manage environmental variables along railway lines to increase the diversity of birds, especially taxonomic and phylogenetic components of their diversity. The most important factors seem to be the presence of bushes/trees, wetlands and wet meadows next to railways. These variables are generally known as important landscape components enhancing bird abundances and species richness (Heikkinen et al. 2004; Riffell et al. 2003). The good conditions/quality of these habitats as well as the continuous loss of these habitats cause all their remnants to be important for birds. Further, the presence of buildings appeared to improve bird diversity. Rural buildings, especially old houses and farmsteads, provide food, shelters and breeding sites for many farmland birds, as was recently observed in Central Europe (Rosin et al. 2016; Šálek et al. 2018). The fields and dry meadows decreased bird diversity. This result is consistent with previous studies that revealed that at dry forest edges, even farmland birds prefer fallow lands to fields (Berg and Pärt 1994; Heikkinen et al. 2004). Surprisingly, geographical direction was also an important factor influencing patterns of bird diversity (Table 3). This result may be linked to the relationship between railways and insulation during the day and thus temperatures that may affect the habitat preferences of birds (Nawaz Pajpar and Zakira 2015).

Our findings on bird diversity components along railways are put in a different context when the conservation status of birds is concerned. At first, it seems that the taxonomic and phylogenetic diversity of rather common, non-threatened species increase along railway embankments compared to control sites (Fig. 3a). Man-made alterations generally lead to the homogenization and trivialization of nature (Marzluff 2001), and previous studies showed that human infrastructure has a neutral effect on more flexible, rather common and broadly distributed species, whereas rare specialists are currently the most negatively affected (Slabbekoorn and Ripmeester 2008). However, in our study, the number of detected decreasing species (IUCN classification) was the same for railway and control transects (*n* = 25). The differences in bird composition between the two landscape types may be due to a higher overall number of species, including six “additional” species with a status of “increasing” along railway transects (17) compared to control transects (11) and two more species with a status of “stable” or “unknown” in the former than in the latter. Thus, this result suggests that railway embankments enhance the diversity of birds, mainly those that are more flexible, but not at the expense of the declining species. Maintaining the landscape mosaic is very important for birds (Heikkinen et al. 2004) and may be important for some rare species associated with open habitats. It may be suspected that the bird diversity indices correlate with IUCN status. However, a review of Pimm et al. (2014) shows that overall and threatened bird distribution does not overlap worldwide. When we repeated analysis testing differences in IUCN status between railway and field transects with the number of species as a covariate, then the number of species was meaningful predictor however was negatively correlated with IUCN status (beta = − 0.018 ± 0.008, *P* = 0.030). The differences between transect types remained statistically significant (beta for railway transects = − 0.174 ± 0.072, *P* = 0.020; intercept = 2.78 ± 0.117).

As the IUCN categories concern the whole distributions of species and may not perfectly capture the circumstances in Poland (relatively low pressure of development and infrastructure in 2009 compared to some other parts of Europe), we decided to also use local measures of population trends. The second measure of “rarity”, with population size estimates for Poland, revealed that there was no difference in species “rarity” between railway and control transects.

### Study constraints

Our study revealed a positive impact of linear structures on bird diversity, but these results should be interpreted with some caution. It should be stressed that this study concerned the impact of railways on bird presence/abundance only and not on their possible breeding success or abundance reduction due to possible collisions. Birds, especially young, inexperienced birds, may be attracted by potential food resources to roadsides that expose them to increased vehicle-related mortality (Erritzoe et al. 2003; Hell et al. 2005). However, the frequency of traffic volume, which is approximately 100 times higher on roads than on railways, and the noise alerting birds seem to be crucial differences between railway and roadway transportation. In Poland, the average traffic volume is 500 vehicles per hour for regional roads (the most frequent type of road) (Opoczyński 2016) and 3–5 trains per hour (source: http://pkpsa.pl). Traffic volume is a main driver of population persistence (Jaeger et al. 2005), and songbirds are able to avoid trains more effectively than they are able to avoid cars (Heske 2015). The effect of railway embankment noise on singing birds and their territories may be lower than that of roads, and the effect of light pollution on moving/migrating birds near railway embankments may be much lower than that near roads (Glista et al. 2009). Moreover, a study on amphibians, which are much poorer dispersers, revealed that they are able to avoid danger in tram tracks (Kaczmarski and Kaczmarek 2016). Therefore, it may be expected that railways may be less harmful than roads, however further research is needed to investigate this problem.

### Management implications

To maintain high species richness, railway embankments should contain a mosaic of bushes, trees and wet habitats. Our results seem to be especially important given the Polish Infrastructure Ministry regulation commanding that all bushes and trees be cleared from railway embankments in 15-m-broad strips (Grabarczyk 2008). This leads to extensive habitat destruction, with 250 000 trees cleared from railway embankments in 2015 alone (Gurgul 2016). The argument of security reasons seems to be misused compared to the far less restrictive standards of other European countries (3 and 5 m in Great Britain and Germany, respectively) with higher train traffic and speed (Anonym 2016). As our study shows, properly managed embankments may be great collateral habitats for a variety of birds. We thus recommend reducing tree and shrub cleaning to only those that are truly dangerous for transportation. On the other hand, the density of shrubs may negatively affect the number and abundance of bees (Moroń et al. 2014). Hence, to preserve the diversity of different groups, it seems reasonable to maintain a mosaic of trees and open habitats (especially wet fragments) along railway embankments.

## Conclusions

This study is recognizing railway embankments as potentially important man-made habitats for bird diversity. We revealed a positive effect of railway embankments on bird community composition and richness and the maintenance of phylogenetic diversity. However, the higher proportion of endangered and declining species recorded along control field transects indicates that the contribution of railways to taxonomic and phylogenetic diversity is achieved through attracting, for the most part, common species that do not increase functionality in agricultural landscapes to a value substantially greater than that in open fields. However, earlier findings that railway embankments are important habitats for other organisms providing ecosystem services (pollinating insects and plants) suggest that this type of man-made habitat may be used to increase habitat heterogeneity and species conservation, which may be achieved through the appropriate management of railway embankments.

## Electronic supplementary material

ESM 1 (DOCX 444 kb)
