# Supplementary material for: The effect of railways on bird diversity in farmland
Source: Environ Sci Pollut Res Int. 2019 Aug 27;26(30):31086–98. doi: 10.1007/s11356-019-06245-0 (PMC6828635; doi:10.1007/s11356-019-06245-0)
Supplement: Supplementary file 1 — (DOCX 444 kb) [file 11356_2019_6245_MOESM1_ESM.docx]

Supplementary material


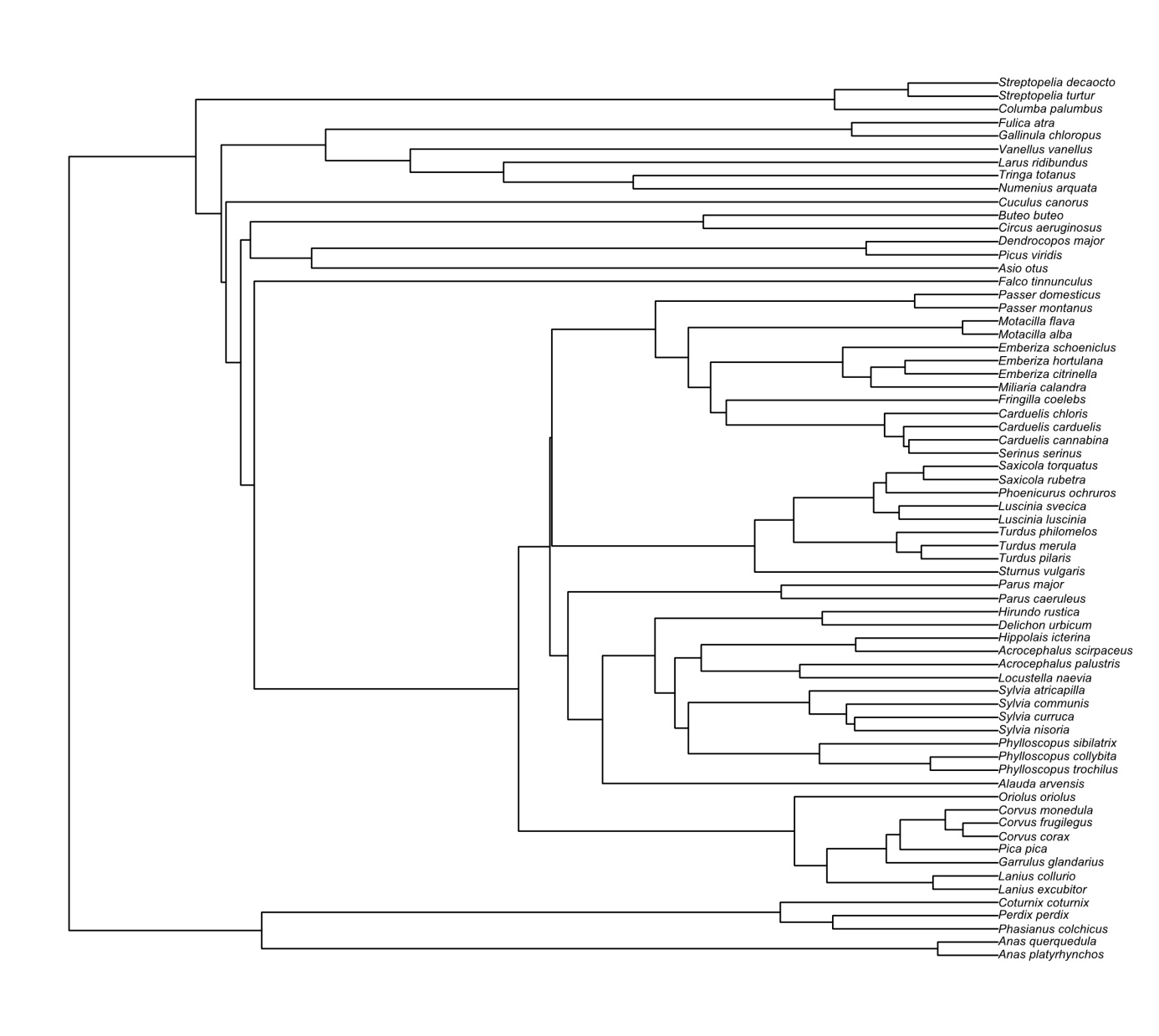


Fig. S1. Phylogenetic tree that was used in analyses.


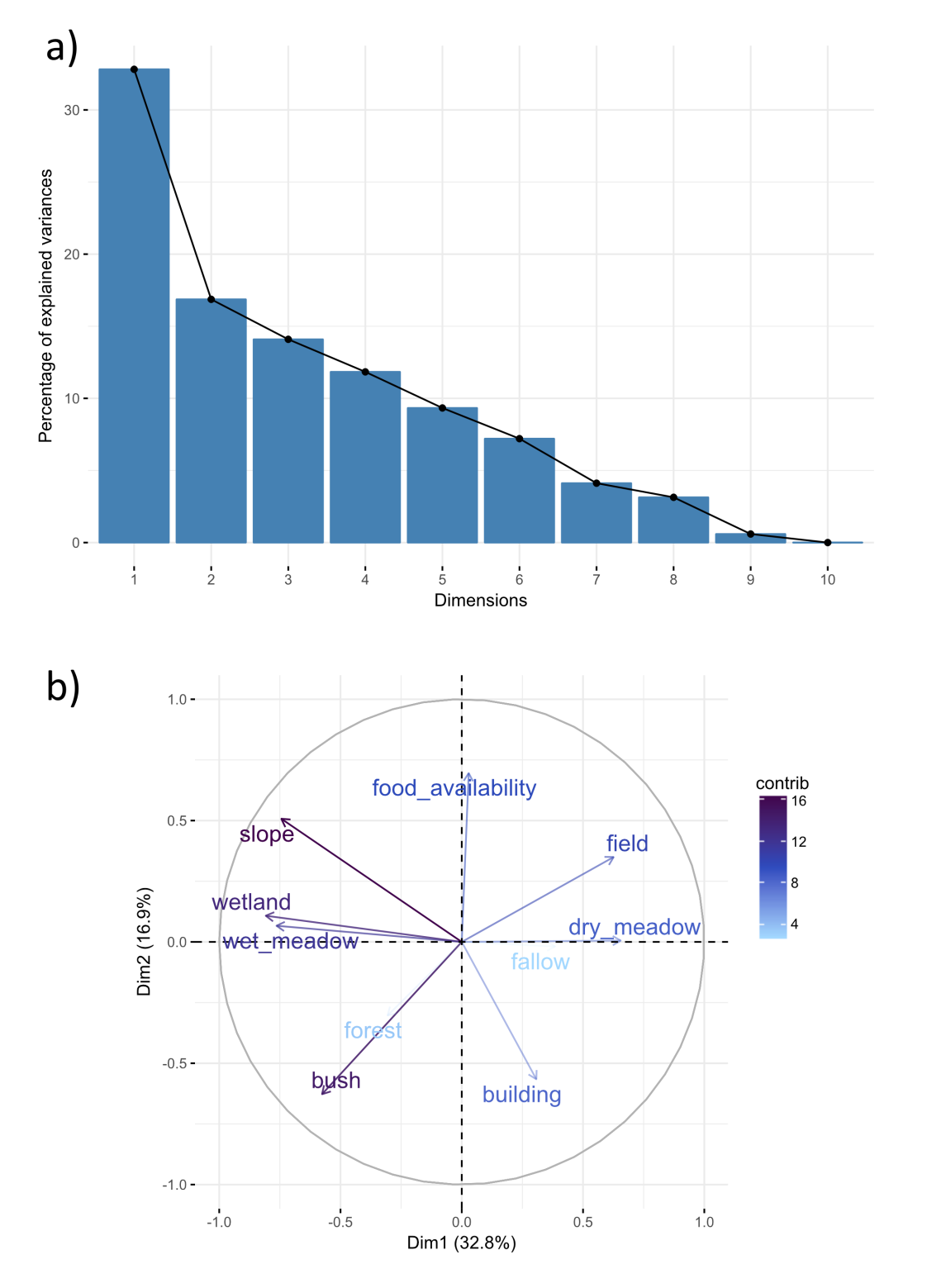


Fig. S2. Results of the principal component analysis (PCA) of continuous environmental explanatory variables used in the analysis of bird diversity indices along railway transects. The variance explained by principal components (a) and correlations between variables along the first two PCA axes (b) are shown. The colours of the environmental variables in the lower panel are scaled based on their percent contribution to the PCA.

Table S1. List of traits with the considered data type and levels of the variable used in functional diversity calculations.

| No | Trait | Type(levels) | Description |
| --- | --- | --- | --- |
| 1 | Brain_mass_g | Continuous | Brain mass [g] |
| 2 | Body_mass_g | Continuous | Body mass [g] |
| 3 | Sexual_dimorphism | Categorical (0 - no, 1 - yes) | Differences between males |
| 4 | Clutch_MEAN | Continuous | Mean clutch size |
| 5 | Broods_per_year | Continuous | Number of broods per year |
| 6 | Egg_MASS | Continuous | Egg mass [g] |
| 7 | Incubation_period | Continuous | Longetivity of incubation period [days] |
| 8 | Fledging_period | Continuous | Longetivity of fledging period [days] |
| 9 | Age_of_first_breeding | Continuous | Age at first reproduction [years] |
| 10 | Life_span | Continuous | Life span [years] |
| 11 | Sedentary | Categorical (0 - no, 1 - yes) | Migration mode: sedentary |
| 12 | Facultative_migrant | Categorical (0 - no, 1 - yes) | Migration mode: facultative migrant (some individuals sedentary) |
| 13 | Short_distance_migrant | Categorical (0 - no, 1 - yes) | Migration mode: short distance migrant |
| 14 | Long_distance_migrant | Categorical (0 - no, 1 - yes) | Migration mode: long distance migrant |
| 15 | Folivore_Y | Categorical (0 - no, 1 - yes) | At least 10% of diet throughout the year composed of grass, leaves, small plants etc. |
| 16 | Frugivore_Y | Categorical (0 - no, 1 - yes) | At least 10% of diet throughout the year composed of fruits |
| 17 | Granivore_Y | Categorical (0 - no, 1 - yes) | At least 10% of diet throughout the year composed of grains, seeds and nuts |
| 18 | Arthropods_Y | Categorical (0 - no, 1 - yes) | At least 10% of diet throughout the year composed of arthropods |
| 19 | Other_invertebrates_Y | Categorical (0 - no, 1 - yes) | At least 10% of diet throughout the year composed of invertebrates except for arthropods |
| 20 | Fish_Y | Categorical (0 - no, 1 - yes) | At least 10% of diet throughout the year composed of fish |
| 21 | Other_vertebrates_Y | Categorical (0 - no, 1 - yes) | At least 10% of diet throughout the year composed of vertebrates except for fish |
| 22 | Carrion_Y | Categorical (0 - no, 1 - yes) | At least 10% of diet throughout the year composed of carrion |
| 23 | Omnivore_Y | Categorical (0 - no, 1 - yes) | Diet throughout the year composed of similar amount of plants and animals |
| 24 | Folivore_B | Categorical (0 - no, 1 - yes) | At least 10% of diet throughout the breeding season composed of grass, leaves, small plants etc. |
| 25 | Frugivore_B | Categorical (0 - no, 1 - yes) | At least 10% of diet throughout the breeding season composed of fruits |
| 26 | Granivore_B | Categorical (0 - no, 1 - yes) | At least 10% of diet throughout the breeding season composed of grains, seeds and nuts |
| 27 | Arthropods_B | Categorical (0 - no, 1 - yes) | At least 10% of diet throughout the breeding season composed of arthropods |
| 28 | Other_invertebrates_B | Categorical (0 - no, 1 - yes) | At least 10% of diet throughout the breeding season composed of invertebrates except for arthropods |
| 29 | Fish_B | Categorical (0 - no, 1 - yes) | At least 10% of diet throughout the breeding season composed of fish |
| 30 | Other_vertebrates_B | Categorical (0 - no, 1 - yes) | At least 10% of diet throughout the breeding season composed of vertebrates except for fish |
| 31 | Carrion_B | Categorical (0 - no, 1 - yes) | At least 10% of diet throughout the breeding season composed of carrion |
| 32 | Omnivore_B | Categorical (0 - no, 1 - yes) | Diet throughout the breeding season composed of similar amount of plants and animals |
| 33 | Precocial | Categorical (0 - no, 1 - yes) | Breeding mode: precocial |
| 34 | Semiprecocial | Categorical (0 - no, 1 - yes) | Breeding mode: semiprecocial |
| 35 | Semialtrical | Categorical (0 - no, 1 - yes) | Breeding mode: semialtrical |
| 36 | Altrical | Categorical (0 - no, 1 - yes) | Breeding mode: altrical |
| 37 | Social_solitary | Categorical (0 - no, 1 - yes) | Sociality during breeding season: teritorial solitary |
| 38 | Social_semi_col | Categorical (0 - no, 1 - yes) | Sociality during breeding season: semi-colonial |
| 39 | Social_colonial | Categorical (0 - no, 1 - yes) | Sociality during breeding season: colonial |

Table S2. Bird species and individuals recorded along railway and field control transects. Species recorded only along railway transects are in bold, and species found only along field control transects are in italics. The conservation status according to IUCN (LC – species of least concern; NT – near threatened species; VU – vulnerable species) coded into the corresponding Category value and the population trend for each species are given.

| Species | Species abbreviation | Railway | Field (control) | Total | IUCN status | Category |  | Population trend | |
| --- | --- | --- | --- | --- | --- | --- | --- | --- | --- |
| Sturnus vulgaris | Stu.vul | 193 | 97 | 290 | LC | 3 |  | decreasing | |
| Alauda arvensis | Ala.arv | 44 | 149 | 193 | LC | 3 |  | decreasing | |
| Sylvia communis | Syl.com | 61 | 11 | 72 | LC | 1 |  | increasing | |
| Hirundo rustica | Hir.rus | 38 | 31 | 69 | LC | 3 |  | decreasing | |
| Motacilla flava | Mot.fla | 24 | 44 | 68 | LC | 3 |  | decreasing | |
| Emberiza citrinella | Emb.cit | 36 | 23 | 59 | LC | 3 |  | decreasing | |
| Miliaria calandra | Mil.cal | 28 | 30 | 58 | LC | 3 |  | decreasing | |
| Saxicola torquatus | Sax.tor | 50 | 5 | 55 | LC | 2 |  | stable | |
| Lanius collurio | Lan.col | 39 | 11 | 50 | LC | 3 |  | decreasing | |
| Passer domesticus | Pas.dom | 17 | 32 | 49 | LC | 3 |  | decreasing | |
| Phasianus colchicus | Pha.col | 22 | 22 | 44 | LC | 3 |  | decreasing | |
| Larus ridibundus | Lar.rid | 36 | 7 | 43 | LC | 4 |  | unknown | |
| Delichon urbicum | Del.urb | 5 | 31 | 36 | LC | 3 |  | decreasing | |
| Columba palumbus | Col.pal | 23 | 11 | 34 | LC | 1 |  | increasing | |
| Turdus pilaris | Tur.pil | 23 | 11 | 34 | LC | 2 |  | stable | |
| Carduelis cannabina | Car.can | 8 | 22 | 30 | LC | 3 |  | decreasing | |
| Pica pica | Pic.pic | 9 | 21 | 30 | LC | 2 |  | stable | |
| Motacilla alba | Mot.alb | 15 | 12 | 27 | LC | 2 |  | stable | |
| Perdix perdix | Per.per | 10 | 15 | 25 | LC | 3 |  | decreasing | |
| Carduelis carduelis | Car.car | 8 | 16 | 24 | LC | 1 |  | increasing | |
| Sylvia atricapilla | Syl.atr | 14 | 7 | 21 | LC | 1 |  | increasing | |
| Phylloscopus trochilus | Phy.tro | 13 | 6 | 19 | LC | 3 |  | decreasing | |
| Sylvia curruca | Syl.cur | 11 | 7 | 18 | LC | 2 |  | stable | |
| Buteo buteo | But.but | 5 | 12 | 17 | LC | 2 |  | stable | |
| Oriolus oriolus | Ori.ori | 14 | 3 | 17 | LC | 2 |  | stable | |
| Cuculus canorus | Cuc.can | 11 | 4 | 15 | LC | 3 |  | decreasing | |
| **Anas platyrhynchos** | Ana.pla | 14 | 0 | 14 | LC | 1 |  | increasing | |
| Carduelis chloris | Car.chl | 9 | 5 | 14 | LC | 2 |  | stable | |
| Coturnix coturnix | Cot.cot | 2 | 12 | 14 | LC | 3 |  | decreasing | |
| Falco tinnunculus | Fal.tin | 5 | 9 | 14 | LC | 3 |  | decreasing | |
| Turdus merula | Tur.mer | 11 | 3 | 14 | LC | 1 |  | increasing | |
| Fringilla coelebs | Fri.coe | 11 | 2 | 13 | LC | 1 |  | increasing | |
| Vanellus vanellus | Van.van | 8 | 5 | 13 | NT | 5 |  | decreasing | |
| Luscinia luscinia | Lus.lus | 7 | 4 | 11 | LC | 2 |  | stable | |
| **Phylloscopus collybita** | Phy.col | 11 | 0 | 11 | LC | 1 |  | increasing | |
| **Emberiza schoeniclus** | Emb.sch | 9 | 0 | 9 | LC | 3 |  | decreasing | |
| Saxicola rubetra | Sax.rub | 5 | 4 | 9 | LC | 3 |  | decreasing | |
| Acrocephalus palustris | Acr.pal | 6 | 2 | 8 | LC | 2 |  | stable | |
| Lanius excubitor | Lan.exc | 4 | 4 | 8 | LC | 3 |  | decreasing | |
| Parus caeruleus | Par.cae | 7 | 1 | 8 | LC | 1 |  | increasing | |
| Streptopelia decaocto | Str.dec | 6 | 2 | 8 | LC | 1 |  | increasing | |
| **Corvus monedula** | Cor.mon | 7 | 0 | 7 | LC | 2 |  | stable | |
| Parus major | Par.maj | 6 | 1 | 7 | LC | 1 |  | increasing | |
| *Passer montanus* | Pas.mon | 0 | 7 | 7 | LC | 3 |  | decreasing | |
| Phoenicurus ochruros | Pho.och | 4 | 3 | 7 | LC | 1 |  | increasing | |
| Emberiza hortulana | Emb.hor | 4 | 2 | 6 | LC | 3 |  | decreasing | |
| **Sylvia nisoria** | Syl.nis | 6 | 0 | 6 | LC | 2 |  | stable | |
| **Turdus philomelos** | Tur.phi | 6 | 0 | 6 | LC | 1 |  | increasing | |
| *Circus aeruginosus* | Cir.aer | 0 | 4 | 4 | LC | 1 |  | increasing | |
| *Corvus frugilegus* | Cor.fru | 0 | 3 | 3 | LC | 3 |  | decreasing | |
| **Gallinula chloropus** | Gal.chl | 3 | 0 | 3 | LC | 2 |  | stable | |
| *Garrulus glandarius* | Gar.gla | 0 | 3 | 3 | LC | 2 |  | stable | |
| **Hippolais icterina** | Hip.ict | 3 | 0 | 3 | LC | 3 |  | decreasing | |
| **Anas querquedula** | Ana.que | 2 | 0 | 2 | LC | 3 |  | decreasing | |
| **Phylloscopus sibilatrix** | Phy.sib | 2 | 0 | 2 | LC | 3 |  | decreasing | |
| **Picus viridis** | Pic.vir | 2 | 0 | 2 | LC | 1 |  | increasing | |
| *Acrocephalus scirpaceus* | Acr.sci | 0 | 1 | 1 | LC | 2 |  | stable | |
| *Asio otus* | Asi.otu | 0 | 1 | 1 | LC | 3 |  | decreasing | |
| **Corvus corax** | Cor.cor | 1 | 0 | 1 | LC | 1 |  | increasing | |
| **Dendrocopos major** | Den.maj | 1 | 0 | 1 | LC | 1 |  | increasing | |
| **Fulica atra** | Ful.atr | 1 | 0 | 1 | LC | 1 |  | increasing | |
| *Locustella naevia* | Loc.nae | 0 | 1 | 1 | LC | 2 |  | stable | |
| **Luscinia svecica** | Lus.sve | 1 | 0 | 1 | LC | 2 |  | stable | |
| *Numenius arquata* | Num.arq | 0 | 1 | 1 | NT | 5 |  | decreasing | |
| *Serinus serinus* | Ser.ser | 0 | 1 | 1 | LC | 3 |  | decreasing | |
| **Streptopelia turtur** | Str.tur | 1 | 0 | 1 | VU | 6 |  | decreasing | |
| **Tringa totanus** | Tri.tot | 1 | 0 | 1 | LC | 4 |  | unknown | |
| Species sum | | 58 | 50 | 68 | - | - | - | |  |
| Abundance total | | 923 | 721 | 1 644 | - | - | - | |  |
